# Invasive meningococcal disease in older adults in North America and Europe: is this the time for action? A review of the literature

**DOI:** 10.1186/s12889-022-12795-9

**Published:** 2022-02-23

**Authors:** Sandra Guedes, Isabelle Bertrand-Gerentes, Keith Evans, Florence Coste, Philipp Oster

**Affiliations:** 1 Sanofi Pasteur, 14 Espace Henry Vallée, 69007 Lyon, France; 2 InScience Communications, Chester, UK

**Keywords:** Atypical Presentation, Clinical Burden, Epidemiology, Invasive Meningococcal Disease, *Neisseria meningitidis*, Older Adults, Recommendations, Serology

## Abstract

**Background:**

*Neisseria meningitidis* is an encapsulated Gram-negative diplococcus that asymptomatically colonises the upper respiratory tract in up to 25% of the population (mainly adolescents and young adults). Invasive meningococcal disease (IMD) caused by *Neisseria meningitidis* imposes a substantial public health burden,. The case fatality rate (CFR) of IMD remains high. IMD epidemiology varies markedly by region and over time, and there appears to be a shift in the epidemiology towards older adults. The objective of our review was to assess the published data on the epidemiology of IMD in older adults (those aged ≥ 55 years)in North America and Europe. Such information would assist decision-makers at national and international levels in developing future public health programmes for managing IMD.

**Methods:**

A comprehensive literature review was undertaken on 11 August 2020 across three databases: EMBASE, Medline and BIOSIS. Papers were included if they met the following criteria: full paper written in the English language; included patients aged ≥ 56 years; were published between 1/1/2009 11/9/2020 and included patients with either suspected or confirmed IMD or infection with *N. meningitidis in* North America or Europe*.* Case studies/reports/series were eligible for inclusion if they included persons in the age range of interest. Animal studies and letters to editors were excluded. In addition, the websites of international and national organisations and societies were also checked for relevant information.

**Results:**

There were 5,364 citations identified in total, of which 76 publications were included in this review. We identified that older adults with IMD were mainly affected by serogroups W and Y, which are generally not the predominant strains in circulation in most countries. Older adults had the highest CFRs, probably linked to underlying comorbidities and more atypical presentations hindering appropriate timely management. In addition, there was some evidence of a shift in the incidence of IMD from younger to older adults.

**Conclusions:**

The use of meningococcal vaccines that include coverage against serogroups W and Y in immunization programs for older adults needs to be evaluated to inform health authorities’ decisions of the relative benefits of vaccination and the utility of expanding national immunization programmes to this age group.

**Supplementary Information:**

The online version contains supplementary material available at 10.1186/s12889-022-12795-9.

## Background

*Neisseria meningitidis* is an encapsulated Gram-negative diplococcus that asymptomatically colonises the upper respiratory tract in up to 25% of the population (mainly adolescents and young adults). Twelve different serogroups cause invasive meningococcal disease (IMD)[1] of which six serogroups (A, B, C, W, X, and Y) are responsible for most infections [2]. *Neisseria meningitidis* is one of the leading causes of bacterial meningitis and sepsis globally [3]; less common presentations include pneumonia and a number of other manifestations [3, 4]. The case fatality rate of meningococcal disease remains high (5–15%) despite treatment [5–7] and survivors can have significant sequelae, with around 20% suffering long-term disability [8]. IMD causes a substantial financial burden, often associated with hospitalisation or ongoing treatment of long-term sequaelae [9–15], as well as negatively impacting the quality of life of patients, their families, caregivers and their extended networks [16, 17].

Vaccination remains the best strategy to prevent IMD [2], and antibiotics are recommended for post-exposure prophylaxis and treatment [18]. IMD is easily misdiagnosed [18–20], because the severity of illness is often obscured by non-specific symptoms [21], and presentation is similar to that of many self-limiting viral infections [22], or there are extra-meningeal foci of infections, including pneumonia, pericarditis, epiglotitis and conjunctivitis [23–25]. There is also a lack of confirmatory testing available in many healthcare settings. Although first-line antibiotics such as third generation cepholosporins are still effective in the treatment of IMD, the emergence of antibiotic-resistant strains have made IMD management more complex. As a result, morbidity and mortality rates have essentially remained unchanged over the last two decades [24, 26–29].

The epidemiology of IMD varies markedly by region and over time but there are an estimated 500,000 newly diagnosed cases per annum [30]. The highest incidences of IMD are found in countries in the African ‘meningitis belt’ region, with the lowest incidences found in parts of Europe and the Americas [3]. A recent systematic review showed that serogroup B was responsible for the highest proportion of *N. meningitidis* IMD cases worldwide; nevertheless, the predominant serogroup varies by region, country, age group and over time [31]. Vaccination against IMD has also contributed to the shift in the predominant serogroups. For example, data from Italy [32], Canada [33] and Germany [34] showed that following the introduction of paediatric meningococcal C vaccination, serogroup C cases in children declined, whilst the median age of those affected by serogroup C increased. For example, in Canada, the median age of cases increased from 16 years in 2003 to 42 years in 2006 [33]. There was also an increased proportion of IMD cases caused by serogroup Y (Germany and Canada) [33, 34] and serogroup B and Y (Italy) [32]. Studies in Australia have also suggested that following the introduction of childhood vaccination against serogroup C, the proportion of notified cases in those aged > 65 years increased [35], whilst studies in the European Union (EU)/European Economic Area (EEA) [36] and Italy [37] also suggested that cases in older adults have increased. Taken together, this would suggest a need to utilise multivalent vaccines and increase vaccine coverage beyond paediatric age groups to counteract these trends.

These epidemiological shifts to older adults have also highlighted the need for further investigation and extension of active surveillance systems (e.g. to include a broader age population than those who are currently covered by national immunisation programs) to accurately assess the changing epidemiology of IMD, and to inform priorities for national health care systems and any associated future vaccination programmes [36–39]. There is an acknowledgement that such data are currently lacking [40].

To date, limited attention has been given to older adults. As such, there is a lack of awareness of the disease in this age group among healthcare professionals, and older adults are not generally considered for immunisation against meningitis. The objective of our review was to assess the published data on the epidemiology of IMD in older adults (generally those aged ≥ 56 years) in North America and Europe to examine how this has changed over time, the impact it has had in terms of clinical burden and mortality, and the extent of currently available data. Such information would assist recommending bodies at national and international levels in developing future public health programmes for preventing IMD.

## Methods

A search was undertaken on 11 August 2020 across three databases: EMBASE, Medline and BIOSIS. The search used MeSH, EMTREE and free text terms as applicable to the databases. Citations were limited to those in English language, in human subjects and published since 1 January 2009. A simplified version of the search strategy is shown in Supplementary Table S1. Papers were included if they met the following criteria: full paper written in the English language (not just the abstract); included patients aged ≥ 56 years; was published after 1 January 2009 but before 11 August 2020; and included patients with either suspected or confirmed IMD or infection with *N. meningitidis in* North America or Europe*.* Case studies/reports/series were eligible for inclusion if they included persons in the age range of interest. Animal studies, non-English language articles and letters to editors were excluded. Review papers were checked to see if they reported primary data or included studies not captured by the database searches, in which case the original papers were ordered and considered for inclusion.

Three authors (KE, SG and PO) assessed the studies independently and discussed any papers for which there were disagreements as to their potential inclusion or exclusion. Data from studies which met the inclusion criteria were then entered into Microsoft Excel. Because the potential studies did not involve standardised study designs and the interventions and comparators were not relevant, only participant data and outcomes data were entered. Because the studies covered a wide range of countries and time periods and used various different methods to determine levels of IMD infection, it was felt that any attempt to combine studies in a formal meta-analysis would not be appropriate; therefore, the data extracted from the studies are discussed in a narrative format.

Additionally, the websites of the following international and national organisations and societies were also searched for relevant data on IMD in the age groups of interest: World Health Organization (WHO); European Centre for Disease Prevention and Control (ECDC); US Centers for Disease Control and Prevention (CDC); Active Bacterial Core Surveillance (ABCs); Emerging Infections Program Network; MenAfriNet; National Foundation for Infectious Diseases; Health Protection Scotland (HPS); Public Health England (PHE); National Institute for Health and Care Excellence (NICE); Institut Pasteur; Robert Koch Institut; Meningitis Research Foundation (MRF) & Meningitis Progress Tracker; Confederation of Meningitis Organisations (CoMO); Meningitis Now; Global Meningitis Genome Library; Infectious Diseases Society of America (IDSA); European Society of Clinical Microbiology and Infectious Diseases (ESCMID); International Society for Infectious Diseases (ISID); American Society for Microbiology (ASM); and European Society for Paediatric Infectious Diseases (ESPID).

## Results

**Fig. 1:**
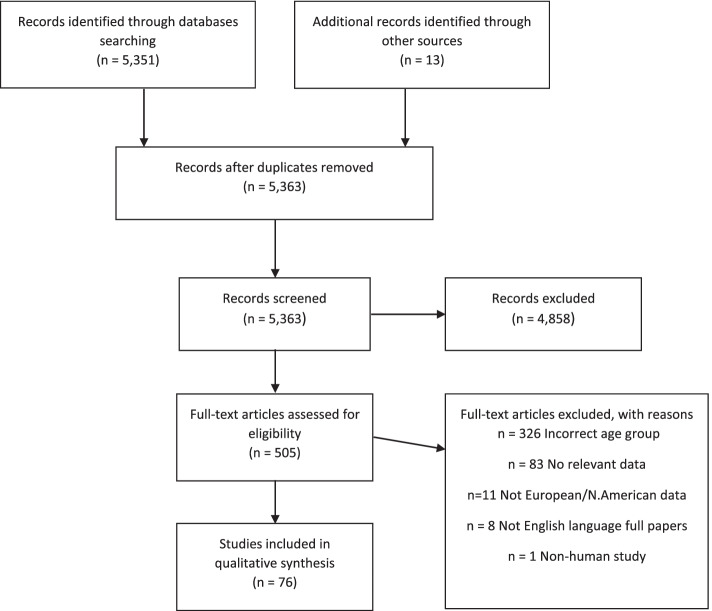
PRISMA Flow Diagram

There were 5,351 citations identified. Following initial review, 505 papers (9% of the original search) plus 13 identified by searching the reference lists of these papers were obtained for full assessment. Following discussion among the authors, a total of 76 papers were included in this review. The reasons for exclusion are summarized in Fig. 1. Data extracted from each study/website was categorised within three headings, as containing data on epidemiology, atypical presentation or clinical burden of IMD (some contained data in multiple categories). Summary information on the published studies included (not including data taken from websites) can be found in Table 1**.** Publications we identified, which reported data from national or international organisations (e.g. CDC or ECDC), were not included in Table 1 if the organisation’s website provided more recent data which we have then presented below.

**Table 1:** Published studies/conference presentations included in the review

Publication	Study Dates	Country	Population	Meningitis confirmation	Key results
Global Burden of Disease Study 2018 [41]	1990–2016	Worldwide	All	Varies by country	Death rate and incidence increased, as did years of life lived with disability (YLD), in the oldest age groups (age groups up to > 95 years), with other meningitis and meningococcal meningitis causing most of the burden in those aged ≥ 80 years. Meningococcus was the leading cause of meningitis mortality in 1990 (192,833 deaths [95% UI 153,358–221,503] globally), Globally in 2016, 1.48 million (1.04–1.96) YLDs were due to meningitis compared with 21.87 million (18.20–28.28) disability-adjusted life-years (DALYs)
Gray et al. 2019 [42]	1998–2019	UK	525–2573 cases per annum	Notified cases to PHE. Mix of PCR test, culture test and PCR/culture testing	The age profile of meningococcal disease cases altered in 2017/18, with an increased proportion of cases in those aged ≥ 45 years. This was subsequent to increases in serogroup W and Y cases, together with the decrease in serogroup B disease
Stefanelli et al. 2015 [43]	1994–2014	Italy	174 IMD cases, out of 4,263 nationally reported (www.iss.mabi), occurred in Puglia	PCR or antisera	Since 2013, 52% of the IMD cases occurred among patients aged ≥ 45 years. The CFR in those aged ≥ 65 was 19%
Säll et al. 2017 [44]	1995–2012	Sweden	A total of 191 patients with serogroup Y IMD were identified in Sweden during the 1995–2012 study period. Of the 191 known episodes of serogroup Y IMD during the study period, medical records for 175 (92%) patients were retrospectively and systematically reviewed. For technical reasons, 16 medical records could not be found, the majority from 1995 to 1999. The median age of the 175 patients in the study was 62 years, and two distinct age groups, 11–20 years and patients > 60 years, together represented the majority of cases (73% of all patients). Four patients were < 5 years of age and only one was < 1 year. Meningitis was diagnosed in 33% and pneumonia in 19% of all patients	Lab confirmed	Two distinct age groups, 11–20 years and > 60 years, together represented the majority of cases (73% of all patients). This age distribution reflects the change in meningococcal epidemiology in Sweden where IMD now largely affects the elderly, with serogroup Y predominating
Folaranmi et al. 2017 [45]	Evaluation of data from January 2012-June 2015	USA	Incidence of MSM in the USA	Meningococcal disease data from the National Notifiable Disease Surveillance System	Within the oldest age group (56–64 years) there was just 1 case out of a total of 527 total cases
***Serology***					
Edge et al. 2016 [46]	2007–2011	UK	IMD cases confirmed by PHE were linked with national hospital records and death registries	Clinical presentation by interrogation of ICD-10 codes	Atypical clinical presentations, including pneumonia and septic arthritis, mainly occurred among those aged ≥ 65 years, caused mainly by serogroups W and Y
Campbell et al. 2020 [47]	2014	UK	340 laboratory-confirmed IMD cases caused by serogroups: B (179 cases), W (95 cases) and Y (66 cases) in individuals aged ≥ 5 years	There were 184 (54%) cases confirmed by culture only, 110 (32%) by PCR only and 46 (14%) by both methods	CFR varied by serogroup and increased with age group, but no significant associations were identified in the multivariable logistic regression models. However, older adults and those with serogroup Y disease were significantly and independently more likely to develop meningococcal pneumonia
Ortiz de Zárate et al. 2016 [48]	1995–2014	Spain	675 invasive *N. meningitidis* isolates were analysed during the study period	Lab confirmed isolates	Serogroup Y isolates were the most frequent among the elderly aged ≥ 65 years
Parisi et al. 2019 [49]	2011–2017	Italy	IMDs surveillance data from the Italian National Health Institute	Presumably notified cases but not explicitly stated	The overall IMDs incidence increased from 0.25 cases/100,000 inhabitants in 2011 to 0.33 in 2017. The increased number of cases in adults and elderly was mostly due to serogroups C, W and Y
Stoof et al. 2015 [50]	1999–2011	The Netherlands	A retrospective study using Dutch surveillance data on IMD from June 1999 to June 2011. Clinical information was retrieved from hospital records. Between June 1999 and June 2011, the NRLBM received 939 isolates from the nine sentinel laboratories. Hospital records were retrieved from 879 (94%) of these IMD cases	Lab confirmed	The overall CFR in this study was 8% and higher for adults compared with children with a clear peak in patients aged ≥ 65 years
Loenenbach et al. 2019[51]	2015–2018	The Netherlands	A total of 565 IMD cases were reported	Lab confirmed	In patients aged ≥ 65 years, CFR overall for this age group was 8.2% (12/146). In patients aged 20–64 years, the CFR overall was 10.2 (18/176)
Eriksson et al. 2018[52]	January 1995-June 2017	Sweden	*N* = 89 IMD cases at the National Reference Laboratory for *Neisseria meningitidis*	Whole genome sequencing	In recent years, a significant increase in the incidence of serogroup W has been noted in Sweden, to an average incidence of 0.15 case/100,000 population in 2015 to 2016. In 2017 (1 January to 30 June), 33% of IMD cases (7/21 cases) were caused by serogroup W
Bennett et al. 2019 [53]	1996/1997 and 2015/2016	Ireland	3,707 cases were reported	National surveillance data on laboratory-confirmed cases	CFR was highest in patients aged > 65 years (15.7%; RR 3.73, 95% CI 2.25–6.19; *P* < 0.0001), although the incidence of IMD was one of the lowest in that age group
Bijlsma et al. 2014 [54]	1998–2002 compared with 2002–2012	The Netherlands	A total of 814 patients were included for analysis	Patients from NRLBM	A figure within the publication breaks out the only data for patients 55 years old and above (data presented for reported cases over the whole study period) and presents an overall incidence rate of approximately 0.2 in those aged 55 years rising to approximately 0.7 in those aged ≥ 90 years
Clarke & Mallonee. 2009 [55]	1988–2004	USA	Cases from the state-wide passive reporting system with disease onset between 1988 and 2004 were included	Passive surveillance	In the ≥ 65-year-old age group, 545 cases of IMD occurred in Oklahoma; 71 (13.0%) died. In those aged > 40 years, serogroup Y was most common (54.6%) followed by B, C and W-135
Peruski et al. 2014 [56]	1988–2011	USA	1,258 cases of IMD were reported to MDPH	Lab confirmed	Throughout the 24-year time period between 1988 and 2011, serogroup Y became increasingly predominant in IMD cases in those aged ≥ 60 years, accounting for over 50% of all serogroup isolates in this age group after 1995
Baccarini et al.2013 [57]	1945–2010	United States and Canada	Review of different studies over the last half century	Varies by study	The distribution of IMD by age was similar in both the USA and Canada. Serogroup Y was proportionally more frequent in adults aged > 65 years in both countries, accounting for over 50% of IMD cases in this age group
Perea-Milla et al. 2009 [27]	1995–2000	Spain	848 patients diagnosed with IMD from 1995 to 2000 in Andalusia and the Canary Islands, Spain	ICD code and some PCR confirmed	A total of 323 patients (38.1%) had sepsis, 336 (39.6%) meningitis and the rest a mixed clinical form, with mortality rates of 10.7%, 2.1% and 4.2%, respectively (*P* < 0.001). Sepsis vs. the other clinical forms had an OR for death of 4. The results showed that that the older the patient, the greater the mortality, with 4.9% deaths in patients aged under 11 years vs. 25% deaths in those aged > 65 years
Gil-Prieto et al. 2011 [58]	1997–2008	Spain	Total of *n* = 6,131 cases	036.0 meningococcal meningitis code	The CFR increased dramatically with age in meningococcal infection, meningococcal meningitis and meningococcemia (*P* < 0.001) reaching the highest values in the > 85-year-old group with 37.66% (95% CI: 26.84–48.49), 42.42% (95% CI: 25.56–59.29) and 37.14% (95% CI: 21.13–53.15) for meningococcal infection, meningococcal meningitis and meningococcemia, respectively
Cabellos et al. 2009 [59]	1977–2006	Spain	Prospective study at a 1,000 bed teaching hospital in Barcelona, Spain. Since 1997, all episodes of community-acquired bacterial meningitis were recorded for cases occurring in patients ≥ 65 years old and these were compared with community-acquired bacterial meningitis occurring in those aged < 65 years	Lab confirmed	There were 675 episodes of meningitis in adults (aged ≥ 18 years) recorded. Of these, 185 (27%) were aged ≥ 65 years (range, 65–93 years). In general, bacterial meningitis in those aged ≥ 65 years was more difficult to diagnose because of the absence of meningeal signs, but the disease had greater neurologic severity and higher rates of complications and mortality
Goldacre & Maisonneuve. 2013 [60]	1999–2010	UK	19,113 people admitted to hospital for meningococcal disease	all people with a discharge diagnosis in HES of meningococcal disease (code A39 in the 10th revision of the ICD, code 036 in the 9th revision) from 1999 to 30 September 2010	OR of CFR by age group 55–59 years5.66 60–64 years5.11 65–69 years8.98 70–74 years6.75 75–79 years8.08 > 80 years6.92
Parent du Chatelet et al. 2017 [61]	2011–2015	France	5,690 cases were biologically confirmed. For 85 (1.5%) cases, the confirmation technique was not reported despite an available group result	Lab confirmed	The CFR was higher in adults ≥ 60 years olds (20%) than in the other age groups (9.9% in infants, 8.9% in 1–4 year-olds, 5.9% in 5–14 year-olds, 9.3% in 25–59 year-olds
Bai et al. 2019 [62]	2019	Eastern Europe but relevant age-specific data for Poland	Findings from Global Round Table Initiative in East Europe	In the Eastern European countries participating in the meeting, the predominant serogroups were serogroup B (accounting for approximately 60–90% of cases) and serogroup C (re-emerging in a number of countries and accounting for up to 30% of cases), followed by serogroup A	CFRs ranged from approximately 3% to 30% both within and across the Eastern European countries represented. In Poland, the greatest CFR (44%) was noted in individuals aged > 65 years
Skoczyńska et al. 2013 [63]	2013 paper analysing data from 2002–2011	Poland	Invasive meningococcal data collected between 2002 and 2011 in the National Reference Centre for Bacterial Meningitis	The isolates were re-identified and characterised by susceptibility testing, MLST analysis, porA and fetA sequencing. A PCR technique was used for meningococcal identification directly from clinical materials	The general CFR was 10.0% for cases with known outcome only, and was highest in patients aged > 65 years (46.2%, *P* = 0.001), although the incidence of IMD was lowest in that age group. Although not broken down by age, the highest CFR was found in patients with sepsis (22.4%), as compared with patients with meningitis and sepsis (7.0%, *P* = 0.0007) and with meningitis alone (3.1%, *P* < 0.0001)
Beebeejaun et al. 2020 [64]	2020 paper based on 2008–2015 data	England	Analysis of surveillance data of laboratory-confirmed IMD cases diagnosed 2008–2015 matched to death registrations	Lab confirmed	In older adults aged ≥ 65 years, all 114 and 134 deaths within one and seven days after diagnosis were IMD-related, as were 96% (146/152) of deaths within 30 days of diagnosis. More than half of the IMD-related fatalities amongst serogroup W cases (44/84, 52%) were in those aged ≥ 65 years and 47/70 (67%) of the IMD-related fatalities amongst serogroup Y cases were in those aged ≥ 65 years
Knol et al. 2017 [65]	2017 paper using surveillance data from 1992–93 to 2015–16	The Netherlands and England	Observational cohort study using surveillance data for the Netherlands and England	Lab confirmed	In the Netherlands, the incidence of meningococcal serogroup W disease increased substantially in 2015–16 compared with 2014–15, with an incidence rate ratio of 5·2 (95% CI: 2·0–13·5) and 11% case fatality. In England, the incidence increased substantially in 2012–13 compared with 2011–12, with an incidence rate ratio of 1·8 (95% CI: 1·2–2·8)
Masson-Behar et al. 2017 [66]	2011–2016	France	A 5-year retrospective study. Included all patients with inflammatory joint symptoms and proven meningococcal disease. A total of 7 patients (5 males) with joint symptoms and meningococcal disease were identified. Of these, 2 had meningitis	Identification of *Neisseria meningitidis* in blood, cerebrospinal fluid, or synovial fluid	Patients presented initially with arthritis
Cikirikcioglu, et al. 2017. [67]	2017	Switzerland	A 55-year-old woman with a history of high fever was admitted to the centre and hospitalized with the diagnosis of bronchopneumonia. Transthoracic echocardiography showed severe aortic valve regurgitation with a mobile vegetation and abscess cavity underneath the left main stem	PCR test	Patient presented with endocarditis
Bajaj et al. 2019 [68]	Not explicitly stated. Year of publication was 2019	USA	A 61-year-old woman with past medical history of diabetes and hypertension presented with fever, chills and headache of 1 day duration	Lab confirmed	This was the fourth case of intraventricular empyema reported secondary to *Neisseria meningitidis*
Keeley et al. 2018 [69]	2018	UK	A 74-year-old Caucasian woman with no history of immunosuppression or rheumatological disease, but with a history of paroxysmal atrial fibrillation for which she was taking flecainide but no anticoagulation, was admitted following a Baltic cruise holiday	PCR confirmed	Patient presented as myopericarditis
Walayat et al. 2018 [70]	2018	USA	The case of a 72-year-old man with a past medical history of severe COPD, obstructive sleep apnoea, and stage I lung cancer status post-stereotactic body radiation therapy 1 year ago	PCR confirmed	Patient presented with a 6-day history of productive cough with yellowish sputum, shortness of breath, extreme myalgia, and fatigue
Romero-Gomez et al. 2012 [71]	2011	Spain	A 94-year-old man sought medical care for left-sided chest pain and difficulty in breathing that began 1 day before admission. He had been healthy until 4 days before admission, when he had sore throat, rhinorrhoea, mild cough, and muscle pain. He had a medical history of ischemic cardiopathy	By the VITEK NHI Identification card and by matrix-assisted laser desorption/ionisation time-of-flight mass spectrometry	Patient presented with bacteraemic pneumonia
Singh & Swann. 2013 [72]	2013	UK	A 55-year-old female nonsmoker with meningococcal septicaemia was treated in an intensive care unit for 11 days, requiring assisted ventilation and renal dialysis. She developed several lesions of necrosis affecting the skin on her arms and legs, as well as ischemic necrosis to her fingers and feet	Not specified in paper	Patient recovered with no requirement for amputation following antibiotic treatment of IMD
Arnáiz-García, et al. 2017 [73]	2017	Spain	A 78-year-old diabetic woman was admitted to the institution presenting fever, diarrhoea, vomiting, and abdominal pain. The patient reported the appearance of red and purplish macules over her lower extremities within the last 4 h	Gram-negative diplococcic, later reported as *Neisseria meningitidis*, was isolated from blood and cerebrospinal fluid cultures	She presented leucocytosis, thrombocytopenia, renal insufficiency, acidosis, and hypoxia cutaneous lesions evolved to haemorrhages and ecchymosis in both hands and feet
Zimmermann & Chmiel 2018 [74]	2018	Switzerland	A 78-year-old woman with a known history of hypertensive cardiomyopathy and paroxysmal benign positional vertigo presented to the emergency department with a 5-day history of throat pain and hoarseness, as well as a progressive and dolorous swelling in the submandibular area	Confirmed by lab test	Patient presented with acute epiglottitis
Rosenfield et al. 2017 [75]	Not explicitly stated. Year of publication was 2017	Canada	A 56-year-old Caucasian woman with past history of severe *Neisseria meningitides* meningitis and bacteraemia at age 42 years, presented with a 2-day history of feeling unwell with vomiting and loose stools	Lab confirmed	Patient presented with terminal complement deficiency
Lesourd et al. 2018 [76]	2018	France	A 85-year-old man was allocated to the emergency department based on an initial fever at home. His previous history included atrial fibrillation, renal lithiasis and benign prostatic hyperplasia	Lab confirmed	Patient presented with primary bacterial ventriculitis
Lawler et al. 2019 [77]	2015	UK	In 2015, two cases of serogroup W IMD occurred in residents aged > 85 years at a 46-bed elderly care home in North East England over a 7-month period; both cases had single bedrooms	*N. meningitidis* was isolated from blood cultures collected on admission	Case 1 was admitted to hospital with acute respiratory distress and fever (temperature 41 °C). Case 2 was admitted to hospital with acute onset of fever, tachycardia and hypotension and was treated for respiratory sepsis unsuccessfully
Puleston et al. 2012 [78]	2012	England	5 people involved in an outbreak in a healthcare setting	Lab confirmed	The 3 confirmed cases all presented with respiratory symptoms
Russcher et al. 2017 [79]	2017	The Netherlands	A man in his early 60 s consulted his general practitioner (GP) because of a painful, red and swollen ankle	Confirmed by tissue culture	Patient presented with necrotising fasciitis
Ladhani et al. 2012 [80]	2007–2009	England and Wales	34 cases in 2007 to 44 in 2008 and 65 in 2009	Lab confirmed	There were 162 laboratory-confirmed W-135 cases reported during 2006–2012 (of which about 44% occurred in those aged > 45 years). Most serogroup W-135 infections in older adults presented as pneumonia (usually in the presence of comorbidities). Fatalities occurred in 5.5% of these cases, all in adults older than 45 years. Based on the graph presented, the CFR would appear to be > 0.1 in those aged 45–64 and < 0.2 in those aged ≥ 65 years
Ristic et al. 2012 [81]	2000–2009	Serbia	There were 94 registered cases	Laboratory confirmed in 34% (32/94) persons	The CFR in this period was 13.8% (septicaemia 26.1%; meningitis 2.1%). The data for meningitis was only reported by two age groups (< 14 years of age and ≥ 15). Of the 48 cases of meningitis, only 12 were in the older group but the age range was not stated. There was one fatality in those aged ≥ 15 years for a CFR of 8.3% (compared with an overall CFR in meningitis of 2.1% for all ages combined)
***Clinical burden***					
Pellegrino et al. 2014 [82]	1993–2011	USA	Two large inpatient databases	Estimated the number of cases of meningococcal meningitis and other bacterial meningitides	The incidence of hospitalisations for meningococcal meningitis (estimated from the publication) were approximately 5 per 100,000 in the 45–64-year age group; 8 in the 65–84-year age group, and 12 in the ≥ 85-years age group

*CFR* Case Fatality Rate, *CI* Confidence Interval, *COPD* Chronic Obstructive Pulmonary Disease, *DALY* Disability-Adjusted Life Years, *HES* Hospital Episode Statistics, *ICD* International Classification of Diseases, *IMD* Invasive Meningococcal Disease, *IQR* Interquartile Range, *MDPH* Massachusetts Department of Public Health, *MLST* Multilocus Sequence Typing, *MSM* Men who have Sex with Men, *NRLBM* Netherlands Reference Laboratory for Bacterial Meningitis, *OR* Odds Ratio, *PCR* Polymerase Chain Reaction, *PHE* Public Health England, *RR* Risk Ratio, *UI* Uncertainty Intervals, *YLD* Years Lived with Disability

### Epidemiology

#### Incidence & Prevalence

A number of studies/websites showed that, over time, an increasing proportion of IMD cases were in older adults, which may in part be because of improvements in surveillance programmes and as a consequence of meningitis vaccination campaigns focusing almost exclusively on infants and adolescents. The Meningitis Progress Tracker [83] estimated that the global number of IMD cases had slowly increased in the period 2000–2017 (the latest year reported) in those aged 25–64 years (from 61,760 in 2000 to 72,430 in 2017) but remained relatively stable in those aged ≥ 65 years (from 2,469 in 2000 to 2,422 in 2017). According to recent estimates published as part of the Global Burden of Disease Study [41], the death rate, years of living with a disability (YLD) rate and incidence all increased in the oldest age groups, with meningococcal meningitis and ‘other’ meningitis causing most of the burden in those aged ≥ 80 years. It should be noted that both the Meningitis Progress Tracker and the Global Burden of Disease Study take estimates from the same sources available via the Institute of Health Metrics and Evaluation (IHME).

Data from Europe, available on the ECDC website for IMD [84], showed that whilst the overall numbers of confirmed IMD cases decreased in the EU/EEA area since 1999, the proportion of cases in those aged > 50 years rose markedly from just under 9% in 1999 to 32% in 2018 (the last year for which data are available). This may reflect the success of the meningococcal vaccination program which focused largely on the youngest age groups, shifting the relative burden to older age groups. Studies from several European countries including the UK [42], Finland [85], Italy [43] and Sweden [44] have confirmed the shift in distribution of IMD cases towards older age groups. For example, Stefanelli et al. showed that 52% of the IMD cases occurred among patients aged ≥ 45 years since 2013 [43].

Data from the US CDC [86] for 2018 showed that the IMD incidence rates rose with increasing age from 0.16 per 100,000 in those aged 55–59 years to 0.49 per 100,000 in those aged ≥ 85 years, and those aged > 45 years accounted for 47% (153/329) of confirmed and probable IMD cases reported that year. Of note, only a single US study of men who have sex with men (MSM) was found despite this being a high-risk group for meningococcal disease [45]. Among 74 cases among MSM (0.56 per 100,000) reported to the National Notifiable Disease Surveillance System between January 2012 and June 2015, only a single case was in the older adult age group (56–64 years) (0.008 per 100,000).

#### Serogroups

There were multiple studies/websites that showed infections caused by serogroups W and Y were more common in older adults than in young children and adolescents. According to data from the ECDC [84], the most prevalent serogroup in Europe during the period 1999–2018 was serogroup B (51% of cases and the dominant serogroup in all age groups below 65 years), with serogroups W and Y increasingly more prevalent in older adults over time. This may reflect the impact of serogroup C vaccinations over this period in teenagers and young adults, with some of the older adults benefitting from ‘herd protection’. A three-fold increase in the incidence of IMD caused by serogroup W was observed between 2013 and 2017, primarily because of increased cases in children aged < 5 years and adults aged ≥ 50 years. This increase in the incidence of IMD caused by serogroup W was confirmed by national institutions such as the Institut Pasteur [87] and many studies in European countries including those from the UK [42, 46, 47], Spain [48], Italy [49], the Netherlands [50, 51], and Sweden [44, 52]. One study in Ireland found serogroup Y as the predominant strain in those aged ≥ 65 years [53]. However, another from the Netherlands reported serogroup B as the most prevalent in older adults rather than serogroups W and Y [54].

Data from the CDC [86] showed that serogroup B was the dominant serogroup in the USA in those aged under 23 years, and serogroups A, C, W and Y were the dominant serogroups in adults and older adults (0.07 per 100,000 and 0.15 per 100,000 in those aged 25–64 years and ≥ 65 years, respectively, compared with 0.03 per 100,000 serogroup B infections in those aged 25–64 years). Latest surveillance data from the ABCs program network that included 10 states reported in 2018 that serogroup Y was more common in adults (aged > 35 years) than in younger adults, in whom serogroups B and C were more frequent [88]. Several US studies also found serogroup Y to be more common in older adults [55–57], for example, Peruski et al. [56] noted that throughout the period 1988 to 2011, serogroup Y became an increasingly predominant cause of IMD in those aged ≥ 60 years, accounting for over 50% of all serogroups isolated in this age group after 1995. One possible explanation for this apparent increase in serogroup Y is that many of the cases in this age group were pneumonia with or without bacteraemia, and the latter are generally not considered to be cases of IMD. Therefore, underreporting of other serogroups may have caused an apparent increase in serogroup Y cases as a proportion of all cases.

#### Mortality

Many of the studies showed that case fatality rates (CFRs) were higher in older adults than in younger adults, adolescents and children. Global data over the period 2000 to 2017 from the Meningitis Research Foundation’s Meningitis Progress Tracker [83] showed that the CFR for those aged > 65 years averaged 12% compared with 7% for those aged 25–64 years.

Data from the ECDC [84] (during the period 1999–2018) showed that the CFR in those aged ≥ 50 years remained relatively stable at 17.4–18.4% from 1999 to 2006 but decreased to approximately 14% in recent years. Multiple published studies in Europe also found higher CFRs in older adults, including those from Spain [27, 58, 59], UK [46, 60, 78], Ireland [53], France [61] and the Netherlands [50]. These studies highlighted that existing comorbidities were an additional risk factor, and combining these with increased age may explain the poorer outcome in older adults. Eastern Europe appears to have much higher CFRs –ranging from approximately 3–46% [62, 63]. However, one single study undertaken in the Netherlands during 2015–2018 found higher CFRs in younger, rather than older, adults [51].

The 2018 surveillance report from the CDC [86] in the USA showed higher CFRs in patients aged ≥ 65 years than that in those aged 25–64 years (23.3 per 100 cases vs 14.1 per 100 cases). The CFRs were higher in these two age groups (25–64 and ≥ 65 years) than in any other age group, including infants (overall average CFR was 12.0 per 100 cases). Other US studies support the higher CFRs observed in older adults [55, 56].

CFRs may also be higher in those affected by serogroups W and Y, which as shown previously are more frequently the cause of IMD in older adults. The 2018 surveillance report from the CDC [86] reported higher CFR associated with serogroup W (23.5 per 100 cases with known outcome) than that for any other serogroup (overall CFR was 12.0%). A similar finding was reported in a US study undertaken for the period 1945 to 2010 by Baccarini et al. [57]. Studies in the UK [64] and the Netherlands [65] have also noted higher CFRs associated with serogroup W and Y cases.

### Atypical clinical presentation

We identified 20 case reports (12 females, 6 males and 2 cases where sex was not reported) from 17 papers [66–77, 79] concerning patients aged ≥ 55 years (range 55–94 years) presenting with atypical symptoms (often linked to a comorbid condition) including myocarditis/endocarditis and arthritis. Half of the total cases identified were patients living in Europe (*n* = 11), 2 cases in North America, and 1 case where the location was not disclosed. In each case, IMD was not initially suspected and *N. meningitidis* was only detected once the patients were admitted to hospital. These case studies highlight the need to be aware of the potential for atypical clinical presentation, to ensure early recognition and treatment and to also allow for susceptibility testing and avoidance of inappropriate antibiotic use and treatment failure. Atypical IMD presentation may be more common in older adults, possibly resulting in delayed diagnosis [47, 78]. In addition, CFRs tended to be higher in those with underlying medical conditions or atypical clinical presentation [27, 57, 63, 80, 81]. There was no mention in any of the case reports of patients having being previously vaccinated.

### Clinical burden

Only two studies that reported resource use in older adults with IMD were identified and both (one in the USA and one in Spain) found an increased incidence of hospitalisation among older adults compared with that in younger adults and adolescents [58, 82].

## Discussion

This comprehensive literature review found evidence that IMD in older adults (those aged ≥ 55 years of age) is mainly caused by serogroups W and Y, which are generally not the predominant circulating strains in any given country or region, and older adults generally have higher CFRs than other age groups (likely linked to underlying comorbidities). Older adults also appear to be more likely to present with atypical symptoms. In addition, there appears to be a shift in IMD prevalence from younger to older people, attributed in part to the success of vaccination programmes against meningitis C in infants and adolescents, but this may also be linked to other factors such as waning immunity amongst those previously vaccinated or immune senescence as a result of aging. Our results are consistent with previous studies which have shown similar trends over time in the epidemiology and clinical presentation of IMD; for example, the link between atypical clinical presentation and higher CFRs [23, 47, 59, 78, 89]. A recent meta analysis of the CFR for laboratory-confirmed IMD cases reported a CFR of 9.0% in infants, which gradually decreased to 7.0% in 7-year olds, subsequently increased to 15.0% in young adults (aged < 28 years), stabilised between 15 and 20% in mid-aged adults and reached a high in older adults [90]. Similar links between age and higher mortality have also previously been reported [4].

There is likely considerable underreporting of IMD cases worldwide. The MRF [83] noted that because meningitis deaths are based on national death registration rather than national surveillance estimates and that because 97% of cases occur in countries with either no or low quality data recording systems, the number of deaths would be substantially underestimated (although it should be noted this comment applies to meningitis in general and not that caused solely by *Neisseria menigitidis*). Improved surveillance systems could help improve disease monitoring. The Global Burden of Disease Study showed that six of the ten countries with the highest number of meningitis deaths (all-causes) are in the African meningitis belt region; though data for older adults from these countries are lacking, suggesting a potential underreporting of cases in older adults in this region [41, 91, 92]. Since we restricted our search to English langauge papers only we decided to exclude date from outside North American and Europe but it is interesting to note that we identified only eleven English language published studies of IMD in older adults from countries outside Europe or North America, which appers to be consistent with previous research highlighting the lack of regional data, particularly from South-East Asia and the Eastern Mediterranean [3] in non-native languages. It is also interesting to note that some of the data from these studies was consistent with the findings from Europe and North America with respect to serology and the shift in the incidence to older age groups [93, 94]. In addition, many of the publications identified in this review, despite having patients in the age group of interest, did not present data on clinical presentation and/or serogroups by age (and indeed when they did present these data, they tended to focus specifically on younger adults) or they presented all meningitis cases together without distinction by pathogenic cause [51, 95–100].

There is evidence to suggest that some risk groups are underrepresented in this review. It is worth noting that IMD cases in MSM are reported as a specific category within the CDC data, and multiple studies in this group have previously been published [101, 102]. There is evidence that younger men are more willing to be vaccinated than older men[103]. However, only a single study in this group was identified in the literature review and only one patient fell within the age range of interest, suggesting the predominant focus is possibly on younger men [45]. It also surprising that, with the exception of two case reports [77], there were no studies examining older adults living in nursing or residential care homes, where one might suspect that close contact between individuals could potentially lead to an increase in transmission of infectious diseases such as IMD.

Of note, the high CFRs observed in older adults in studies included in this review, up to 34% [104], are consistent with those reported in a recent meta-analysis of laboratory-confirmed IMD. In addition, the meta-analysis also showed that CFRs generally increased with age and were highest in the oldest age groups [90]. As such, older adults represent an unmet need for meningococcal vaccination because, as noted by Trezikowski de Lima et al. [105] “given the increasing proportion of older people in the population and the high CFR of meningococcal disease in the elderly, it would be interesting to evaluate the insertion of these vaccines in the immunization programs for this age group…also, vaccines can generate other benefits, e.g. lower overall cost of healthcare”.

The strengths of this study were that it included a comprehensive literature review and grey literature search to supplement the data derived from publications. However, there was relatively little information on IMD in older adults, and the difficulties of interpreting the results were compounded by the inconsistent reporting by age group, or where demographic breakdown of a study population was presented in detail the subsequent results (e.g. epidemiology, clinical presentations) were only presented for the cohort as a whole. The issue is further complicated by the lack of standardised case definitions, changes to national immunisation programmes over time and the varying surveillance and laboratory techniques employed worldwide, as commonly acknowledged [106, 107]. As such, there remains the need for more specific age-related studies and improvements in consistency of reporting across all age groups, including older adults [108, 109].

It should also be noted that the data presented here predate the severe acute respiratory syndrome coronavirus 2 pandemic and the introduction of coronavirus disease 2019 (COVID-19) control measures; social distancing and shielding appear to have led to a decrease in recorded cases of IMD in some countries [110]. However, IMD cases that were associated with respiratory presentations of which some corresponded to suspected COVID-19 appeared to increase in 2020 compared with 2018 (*P* = 0.029) and 2019 (*P* = 0.002) and involved the elderly and with unusual isolates [111]. Moreover, IMD concomitant with COVID-19 may be associated with poorer outcomes in the elderly, because the prognosis of either disease is usually worse in this age group, though definitive data are lacking. Nonetheless, IMD burden would likely return to previous levels once on-going COVID-19 measures are relaxed, and as such continued surveillance for meningococcal and invasive bacterial infections will also be important as the pandemic progresses. The authors of the latter study [111] concluded that surveillance of IMD should be improved and vaccination against meningococcal disease in older adults should be considered (currently only Italy suggests adopting a lifelong approach to vaccination, with regular immunisation being offered to adults in the future) [112].

## Conclusions

This comprehensive literature review, supplemented by data from national organisations, institutions and societies provides evidence that older adults (those aged ≥ 55 years) with IMD are mainly affected by serogroups W and Y, which are generally not the predominant strains in circulation in any country. Older adults have the highest CFRs, probably linked to underlying comorbidities and more atypical presentations hindering appropriate timely diagnosis and management. In addition, there has been a shift in the incidence of IMD from younger to older adults, which may be attributed to the success of meningococcal vaccination programmes, although the exact scale of this shift is difficult to quantify. Future research should evaluate the alternative options of either implementating adolescent vaccination programmes with conjugate vaccines in some countries that may lead to indirect protection in older adults or te use of meningococcal vaccines that include coverage against serogroups W and Y in immunization programs for older adults to help inform health authorities’ decisions of the benefits of vaccination and the utility of expanding national immunization programmes to extend protection to older adults.

## Supplementary Information

**Additional file 1.**

## Data Availability

All data generated or analysed during this study from published articles or conference presentations are included in this published article (Table 1). Any data generated or analysed from international or national organisations is available from the weblinks presented in Supplementary Table 2.

## Abbreviations

ABCs: Active Bacterial Core surveillance
ASM: American Society for Microbiology
CDC: Centers for Disease Control and Prevention
CFR: Case Fatality Rate
95% CI: 95% Confidence Interval
CoMO: Confederation of Meningitis Organisations
COVID-19: Coronavirus disease 2019
ECDC: European Centre for Disease Prevention and Control
EEA: European Economic Area
ESCMID: European Society of Clinical Microbiology and Infectious Diseases
ESPID: European Society for Paediatric Infectious Diseases
EU: European Union
HPS: Health Protection Scotland
IDSA: Infectious Diseases Society of America
IHME: Institute for Health Metrics and Evaluation
IMD: Invasive Meningococcal Disease
ISID: International Society for Infectious Diseases
MRF: Meningitis Research Foundation
MSM: Men who have Sex with Men
NICE: National Institute for Health and Care Excellence
PHE: Public Health England
UK: United Kingdom
USA: United States of America
WHO: World Health Organization
YLD: Years of Life lived with Disability
