# Supplementary material for: Invasive meningococcal disease in older adults in North America and Europe: is this the time for action? A review of the literature
Source: BMC Public Health. 2022 Feb 23;22:380. doi: 10.1186/s12889-022-12795-9 (PMC8864456; doi:10.1186/s12889-022-12795-9)
Supplement: Supplementary file 1 — Additional file 1. [file 12889_2022_12795_MOESM1_ESM.docx]

**Supplementary Table S1: Search strategy**

| **Search line number** | **Search Strategy** | **Number of citations** |
| --- | --- | --- |
| 1 | (((ab((ab((pneumonia or "interstitial plasma cell pneumonia" or "pneumocystosis" or "pneumonias"), bacterial OR (pneumonia or "interstitial plasma cell pneumonia" or "pneumocystosis" or "pneumonias") OR (((lung or "pulmo") diseases interstitial) or ("lung disease" and interstitial) or ("pulmonary disease" and interstitial)) OR (septicemia or "septic fever" or "septic intoxication" OR Sepsis OR Septic* OR Bacteremia OR Bacteremia) OR neisseriaccae (infection or "autoinfection" or "autoreinfection" or "infections" or "infectivity" or "self-infection") OR meningococcossis)) AND ab(Meningococcal OR Neisseria)) and (pd(>20090101)) and (human(yes) AND human(yes) AND human(yes)) and (la.exact("English"))) and (pd(>20090101)) and (human(yes) AND human(yes) AND human(yes)) and (la.exact("English")))) | 1181 |
| 2 | (((haemophil* (influenza or "flu" or "grippe")) OR Haemophilus influenzae type b OR "Hib" OR "group B streptococc*" OR "GBS" OR Streptococcus agalactiae) OR Streptococcus pneumonia* OR Pneumonia, Pneumococcal) | 250641 |
| 3 | (ab(meningococcal (meningitis or "cerebrospinal meningitis" or "meningitides" or "meningitidis" or "pachymeningitides") or "cerebrospinal fever" or "epidemic cerebrospinal meningitis" OR (Neisseria AND (meningitides OR meningitidis OR meningitis)))) and (pd(>20090101)) and (human(yes) AND human(yes) AND human(yes)) and (la.exact("English")) | 6671 |
| 4 | ((ab((ab((pneumonia or "interstitial plasma cell pneumonia" or "pneumocystosis" or "pneumonias"), bacterial OR (pneumonia or "interstitial plasma cell pneumonia" or "pneumocystosis" or "pneumonias") OR (((lung or "pulmo") diseases interstitial) or ("lung disease" and interstitial) or ("pulmonary disease" and interstitial)) OR (septicemia or "septic fever" or "septic intoxication" OR Sepsis OR Septic* OR Bacteremia OR Bacteremia) OR neisseriaccae (infection or "autoinfection" or "autoreinfection" or "infections" or "infectivity" or "self-infection") OR meningococcossis)) AND ab(Meningococcal OR Neisseria)) and (pd(>2009010)) and (human(yes) AND human(yes) AND human(yes)) and (human(yes) AND human(yes) AND human(yes)) and (la.exact("English"))) and (pd(>20090101)) and (human(yes) AND human(yes) AND human(yes)) and (la.exact("English"))) AND adult* NOT ((fetus or newborn* or infant* or preschool or child* or adolescent*)) | 222 |
| 5 | 1 NOT 2 | 918 |
| 6 | ((ab(meningococcal (meningitis or "cerebrospinal meningitis" or "meningitides" or "meningitidis" or "pachymeningitides")) OR ab("cerebrospinal fever" or "epidemic cerebrospinal meningitis") OR ab(Neisseria AND (meningitides OR meningitidis OR meningitis)) OR ab("invasive meningococcal disease" OR "IMD" or "invasive meningococcal") OR mesh(Meningococcal meningitidis OR Meningococcal infections) OR ab("Meningococcal meningitides" OR "Meningococcal infections") AND adult*) and (pd(>20090101)) and (human(yes) AND human(yes) AND human(yes)) and (la.exact("English"))) NOT ab(fetus or newborn* or infant* or preschool or child* or adolescent*) | 7730 |
| 7 | 5 OR 6 | 8487 |
| 8 | 4 NOT 2 | 158 |
| 9 | 7 OR 8 | 7795 |
| 10 | 9 and publication date > 1/1/2009 and duplicates removed | 5351 |

**Supplementary Table S2**

| **Organisation** | **Web address** |
| --- | --- |
| Centers for Disease Control and Prevention: | <https://www.cdc.gov/meningococcal/index.html> |
| Centers for Disease Control and Prevention: Active Bacterial Core Surveillance Report | <https://www.cdc.gov/abcs/reports-findings/survreports/mening18.html> |
| European Centre for Disease Prevention and Control | <https://www.ecdc.europa.eu/en/meningococcal-disease/surveillance-and-disease-data/atlas> |
| Institut Pasteur - Centre National de Reference des Meningocoques | <https://www.pasteur.fr/fr/file/3273/download?token=8UaruHXt> |
| Meningitis Research Foundation – Meningitis Progress Tracker | <https://www.meningitis.org/mpt> |
| Società Italiana di Igiene | <https://www.vaccinarsi.org/notizie/2019/07/%E2%80%8Bcalendario-vaccinale-per-la-vita-2019> |
